# Obesity and Metabolic Dysregulation in Children Provide Protective Influenza Vaccine Responses

**DOI:** 10.3390/v14010124

**Published:** 2022-01-11

**Authors:** Mundeep K. Kainth, Joanna S. Fishbein, Teresa Aydillo, Alba Escalera, Rachael Odusanya, Kalliopi Grammatikopoulos, Tiffany Scotto, Christine B. Sethna, Adolfo García-Sastre, Clifford S. Deutschman

**Affiliations:** 1 Cohen Children’s Medical Center, Department of Pediatrics, Northwell Health, Queens, NY 11040, USA; kgramma1@binghamton.edu (K.G.); tiffanyscotto513@gmail.com (T.S.); csethna@northwell.edu (C.B.S.); cdeutschman@northwell.edu (C.S.D.); 2 Donald and Barbara Zucker School of Medicine at Hofstra/Northwell, Northwell Health, Hempstead, NY 11549, USA; 3 Feinstein Institutes for Medical Research, Northwell Health, Manhasset, NY 11030, USA; jfishbein1@northwell.edu; 4 Department of Microbiology, Icahn School of Medicine at Mount Sinai, New York, NY 10029, USA; teresa.aydillo-gomez@mssm.edu (T.A.); alba.escalera@icahn.mssm.edu (A.E.); adolfo.garcia-sastre@mssm.edu (A.G.-S.); 5 Global Health and Emerging Pathogens Institute, Icahn School of Medicine at Mount Sinai, New York, NY 10029, USA; 6 Graduate School of Biomedical Sciences, Icahn School of Medicine at Mount Sinai, New York, NY 10029, USA; 7 Lewis Katz School of Medicine at Temple University, Philadelphia, PA 19122, USA; rachael.odusanya@temple.edu; 8 Philadelphia College of Osteopathic Medicine, Philadelphia, PA 19104, USA; 9 Department of Pathology, Molecular and Cell-Based Medicine, Icahn School of Medicine at Mount Sinai, New York, NY 10029, USA; 10 Department of Medicine, Division of Infectious Diseases, Icahn School of Medicine at Mount Sinai, New York, NY 10029, USA; 11 The Tisch Cancer Institute, Icahn School of Medicine at Mount Sinai, New York, NY 10029, USA

**Keywords:** pediatric obesity, influenza vaccine, metabolic health

## Abstract

The most effective intervention for influenza prevention is vaccination. However, there are conflicting data on influenza vaccine antibody responses in obese children. Cardio-metabolic parameters such as waist circumference, cholesterol, insulin sensitivity, and blood pressure are used to subdivide individuals with overweight or obese BMI into ‘healthy’ (MHOO) or ‘unhealthy’ (MUOO) metabolic phenotypes. The ever-evolving metabolic phenotypes in children may be elucidated by using vaccine stimulation to characterize cytokine responses. We conducted a prospective cohort study evaluating influenza vaccine responses in children. Participants were identified as either normal-weight children (NWC) or overweight/obese using BMI. Children with obesity were then characterized using metabolic health metrics. These metrics consisted of changes in serum cytokine and chemokine concentrations measured via multiplex assay at baseline and repeated at one month following vaccination. Changes in NWC, MHOO and MUOO were compared using Chi-square/Fisher’s exact test for antibody responses and Kruskal–Wallis test for cytokines. Differences in influenza antibody responses in normal, MHOO and MUOO children were statistically indistinguishable. IL-13 was decreased in MUOO children compared to NWC and MHOO children (*p* = 0.04). IL-10 approached a statistically significant decrease in MUOO compared to MHOO and NWC (*p* = 0.07). Influenza vaccination does not provoke different responses in NCW, MHOO, or MUOO children, suggesting that obesity, whether metabolically healthy or unhealthy, does not alter the efficacy of vaccination. IL-13 levels in MUO children were significantly different from levels in normal and MHOO children, indicating that the metabolically unhealthy phenotypes may be associated with an altered inflammatory response. A larger sample size with greater numbers of metabolically unhealthy children may lend more insight into the relationship of chronic inflammation secondary to obesity with vaccine immunity.

## 1. Introduction

Obesity is becoming increasingly prevalent, now including 1 in 5 children ages 2–18 years old in the United States [1]. Obesity is a risk factor for severe influenza in both children and adults, especially in those who are unvaccinated [2,3,4,5,6,7]. Viral respiratory infections, nasal carriage of pathogenic bacteria, and complications or hospitalizations due to the influenza H1N1 pandemic strain were increased in obese children [8,9,10,11]. It is not clear how obesity affects the response to influenza infection or vaccination. Data are limited on the role of immune compromise in the response to vaccination, and reported results are conflicting [11,12]. Thus, we are investigating a gap in our knowledge regarding the host immune response to influenza vaccination in children with obesity.

Recent studies have sub-divided obese individuals into metabolically healthy overweight/obese (MHOO) and metabolically unhealthy overweight/obese (MUOO) cohorts [13]. The established definition of metabolic health uses standard indices such as blood pressure (BP), waist circumference (WsC), fasting glucose, HbA1c, and triglycerides. Metabolic health may be a mediating factor in obesity that has yet to be identified as a possible cause for poor immune response to influenza disease or vaccination.

There is a lack of sufficient knowledge as to whether influenza vaccination provides adequate seroconversion rates in the pediatric obese population. In particular, obese adolescents are often overlooked in studies as a primary population of interest. This investigation redefines the approach to obesity by evaluating influenza vaccine responses through the lens of metabolic health. Our exploratory study evaluated BMI and the influenza vaccine in metabolically healthy versus unhealthy obese children and used cytokine/chemokine responses to identify phenotypes of immune dysregulation.

## 2. Materials and Methods

We conducted an exploratory prospective cohort study at Cohen’s Children’s Medical Center at Northwell Health during the months of October–March in 2017–2020. This investigation, which required informed consent and was approved by the Northwell Health Institutional Review Board, evaluated influenza vaccine responses in children with normal weight and overweight/obese BMI percentiles between 9 and 17 years of age. Participants who were registered for an influenza vaccination visit or well-child visit. We excluded participants with a history of immunodeficiency, immunosuppressive medications, or BMI < 5th percentile for age even if still eligible for influenza vaccine. We excluded children who already received influenza vaccination during the recruitment season. Vaccination in previous influenza seasons was not a reason for exclusion.

At the baseline visit, demographic and clinical data including height, weight, WsC and BP was obtained. Height was measured to the nearest 0.1 cm with a stadiometer, and weight was measured to the nearest 0.1 kg with a digital scale. WsC was measured at the level of the umbilicus and the superior iliac crest using a flexible tape measure. Measurements were made in triplicate, and the average was used for analysis. Casual BP measurements were obtained in triplicate at each study visit using a calibrated oscillometric device. BPs were measured in the right arm with the participant in a seated position after five minutes of rest. The average of the last two readings was used. The 2017 clinical practice guidelines were used to classify blood pressure percentiles [14]. The Fluzone quadrivalent vaccine (0.5 mL) was administered at baseline visit. Participants had a fasting blood draw prior to vaccination and during a second visit at 25–42 days (approximately 1 month). Blood samples were analyzed at Core Laboratories, Northwell Health.

### 2.1. Case Definitions

We initially identified participants with overweight/obesity by calculating BMI and assigning percentiles. Normal-weight children (NWC) were defined as BMI < 85th percentile. Children with overweight/obesity were defined as BMI ≥ 85th percentile [15]. We categorized children with overweight/obese in MHOO and MUOO cohorts using their BP, WsC, fasting glucose, and triglycerides [16,17,18]. MUOO was defined as having at least 2 of the following 5 cardiometabolic risk factors: (1) WsC ≥ 90th percentile, (2) triglyceride (TG) level ≥ 110 mg/dL, (3) high-density lipoprotein (HDL) level ≤ 40 mg/dL, (4) both systolic and diastolic blood pressure ≥ 90th percentile, (5) fasting plasma glucose ≥ 100 mg/dL. MHOO was defined as having no more than one of the cardiometabolic risk factors.

### 2.2. Influenza Virus Assays

Samples were analyzed at the Mount Sinai School of Medicine for serotype-specific influenza antibody response, which was determined by hemagglutination inhibition (HAI) assay and performed as previously described [19]. For this, serum samples were treated with receptor-destroying enzyme (RDE) (Denka Seiken) according to the manufacturer’s instructions. Briefly, 1 part serum sample was mixed with 3 parts RDE solution (reconstituted with phosphate-buffered saline [PBS]) and incubated for 16–18 h in a 37 °C water bath. RDE was inactivated with 3 volumes (based on original serum volume) of 2.5% sodium citrate solution and then incubated at 56 °C for 30 min. Additionally, 3 volumes of PBS based on original serum volume were added for a final human serum sample concentration of 1:10. Serum samples were assessed for antibodies against 4 influenza virus strains (A/H1N1, A/H3N2, B/Victoria and B/Yamagata lineages) contained in the quadrivalent vaccines according to year of vaccination: 2017-2018 (A/Michigan/45/2015, A/Hong Kong/4801/2014, B/Brisbane/60/2008 and B/Phuket/3073/2013), 2018–2019 (A/Michigan/45/2015, A/Singapore/16/2016, B/Colorado/06/2017 and B/Phuket/3073/2013) and 2019–2020 (A/Brisbane/02/2018, A/Kansas/14/2017, B/Colorado/06/2017 and B/Phuket/3073/2013). Influenza virus strains were diluted to a final concentration of 8 HA units/50 mL in fluorescent treponemal antibody (FTA) hemagglutination (HA) buffer (BD Biosciences). Two-fold dilutions of RDE treated serum were then incubated with equal amount of the virus at 8 HA units/50 mL (30 min, room temperature). Turkey red blood cells (RBCs) (Lampire Biological) at 0.5% in HA buffer (50 mL) were added and incubated for 45 min at 4 °C. The HAI titer was determined by taking the reciprocal dilution of the last well in which serum inhibited the hemagglutination of RBCs. Sera without reactivity were assigned a value of 5 (<10). Sera were considered positive based on international criteria of seroprotection if neutralizing titers measured were ≥40 [20].

We compared changes in titers between baseline and 1 month follow-up visit within groups. Based on previous studies on influenza inactivated trivalent vaccine in obese children, immunogenicity was evaluated using the criteria for adults aged 18–60 years [11,12], which require at least one of the following for each strain:

1. Baseline serologic titer;
2. Seroconversion: a ≥ 4-fold increase in HI antibody titer with a titer of ≥1:40 being reached;
3. Seroprotection: an HI antibody titer of ≥1:40;
4. Geometric mean ratio/titer: The GMR/GMT and 95% confidence intervals were calculated by taking the exponent (log2) of the mean and of the lower and upper limits of the 95% confidence intervals of the log2-fold induction transformed of post-vaccination/pre-vaccination titers. These parameters account for baseline elevation in serologic titers due to previous vaccination or exposure to influenza disease.

### 2.3. Multiplex Cytokine Assay

Samples were sent to Eve Cytokines, Inc. for single analysis of serum concentrations of granzyme B, procalcitonin, adiponectin, leptin, ferritin, RANTES, IL-12p40/p70, GM-CSF, IFNg, MIF, TNFa, MCP-1, IL-1b, IL-2, IL-5, IL-6, IL-8, IL-10, IL-13, IL-17A, and IL-23. These were measured via multiplex assay to identify concentrations in the obese participants and then the subset of MHOO/MUOO participants compared to NWC. The magnitude of the change was identified between baseline and 1 month after vaccination. We compared the percent increase/decrease in serum concentration of each cytokine or chemokine in the normal weight, MUOO, and MHOO populations.

### 2.4. Statistical Considerations

Frequencies and proportions for categorical variables and geometric means and geometric standard deviations for titer levels were computed for the sample overall with complete data and broken down by BMI/metabolic health status classifications. The Monte Carlo estimate for the exact Kruskal–Wallis test was utilized for continuous variables such as CRP, adiponectin, and other cytokine levels that did not meet normality assumptions. Otherwise, analysis of variance (ANOVA) was performed for comparisons of the three groups on continuous data that met assumptions (e.g., to compare the mean differences of the GMTs within each lineage). When appropriate, pairwise comparisons were performed using two-sample *t*-tests, with Dunn’s test for *p*-value adjustment to account for the multiple pairwise comparisons. Chi-square tests or Fisher’s exact tests, when appropriate, were utilized to compare seroprotection and seroconversion rates among the groups. For all analyses, a result yielding a *p*-value < 0.05 was considered statistically significant. All analyses were conducted using SAS version 9.4 (SAS Institute Inc., Cary, NC, USA).

## 3. Results

A total of 79 participants were enrolled in our study. A total of 43 participants provided baseline and 1-month follow-up serum samples. Of the 43 participants, 19 (44%) were identified as overweight or obese. Females were slightly more represented (60%) compared to males, with an equal distribution among race. WsC > 90th percentile, glucose > 100 mg/dL, and triglycerides > 110 mg/dl were frequently present in the cohort, at 13 (30%), 11 (26%), and 11 (26%) of participants, respectively. Elevated BP and low HDL were infrequent 3 (7%). Using our chosen parameters, 7/19 met criteria for MUOO. Interestingly, 3 (7%) participants were normal weight but metabolically unhealthy. (Table 1).

**Figure 1:**
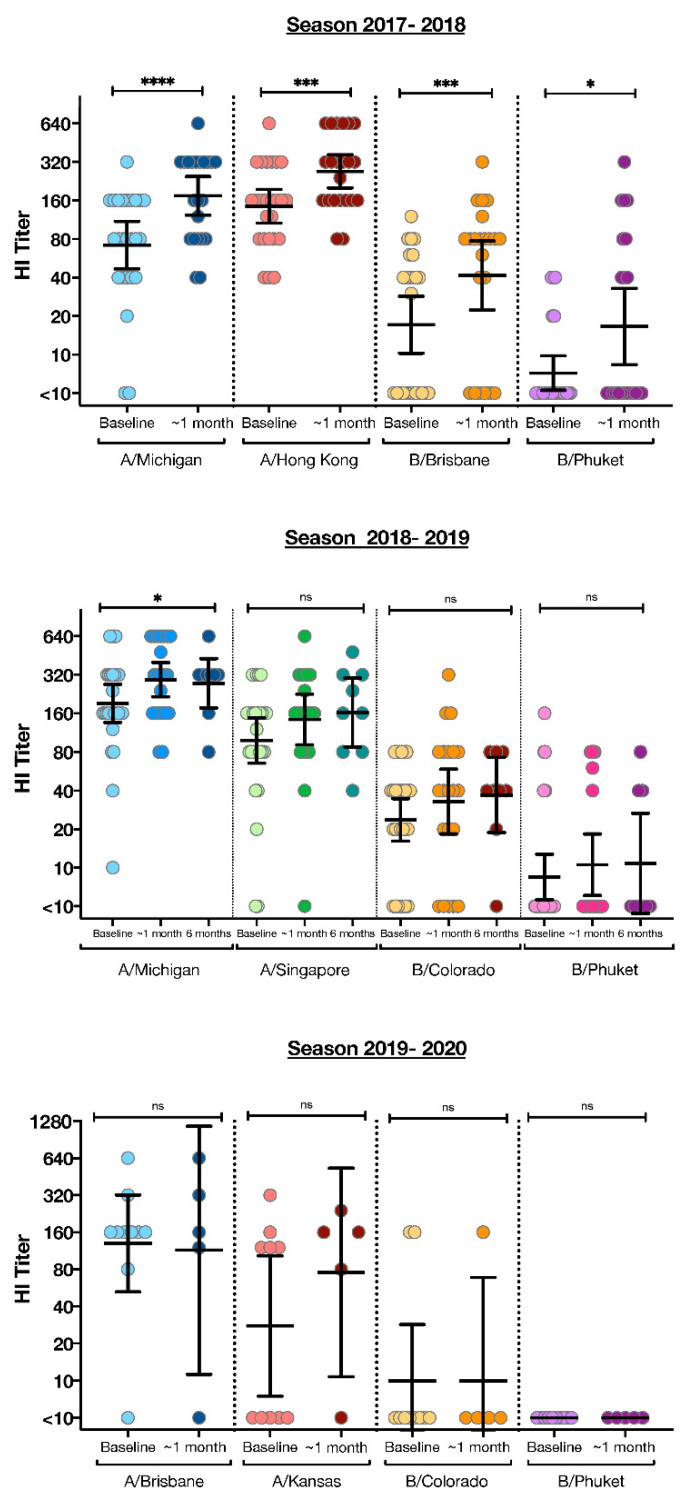
Characterization of influenza responses in the entire cohort of children measured by hemagglutination inhibition (HI) assays. HI titers before vaccination (at baseline), ~1 month, and 6 months after vaccination are shown for each of the vaccine strains contained in the quadrivalent influenza vaccine (Fluzone) for three consecutive seasons (2017–2018, 2018–2019, 2019–2020). **** *p* < 0.0001; *** *p* < 0.001, * *p* < 0.05. ns: indicated Not Significant.

All 43 of our participants had paired visit 1 and visit 2 antibody results for B/Phuket/3073/2013–like virus (Yamagata lineage). A total of 21 participants had paired sera specimens for B/Colorado/06/2017-like virus and 22 participants for B/Brisbane/60/2008–like virus (Victoria lineages). Furthermore, 38 of our participants had antibody responses described for A/Michigan/45/2015 (H1N1)pdm09–like virus and 5 for A/Brisbane/02/2018 (H1N1)pdm09-like virus. For the H3N2 lineage, including A/HongKong/4801/2014 (H3N2)–like virus (*n* = 22), A/Singapore/INFIMH-16-0019/2016 A(H3N2)-like virus (*n* = 16), and A/Kansas/14/2017 (H3N2)-like virus (*n* = 5), a total of 43 paired sera specimens were analyzed (Figure 1). For all strains, there were no significant statistical differences among NWC, MUOO, and MHOO children when evaluating for seroprotection, defined as an HI antibody titer of at least 1:40, seroconversion, defined as a 4-fold increase in HI antibody titer and a titer of at least 1:40, and GMR/GMT compared across the 3 groups (NWC, MHOO, MUOO) (Table 2, Table 3 and Table 4).

**Figure 2:**
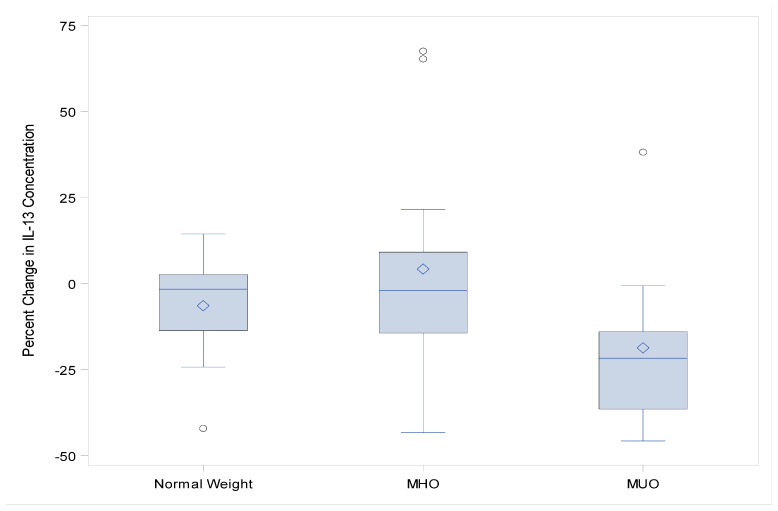
IL-13 percent change in concentration from baseline to 1-month post-influenza vaccination (*p* < 0.04).

**Figure 3:**
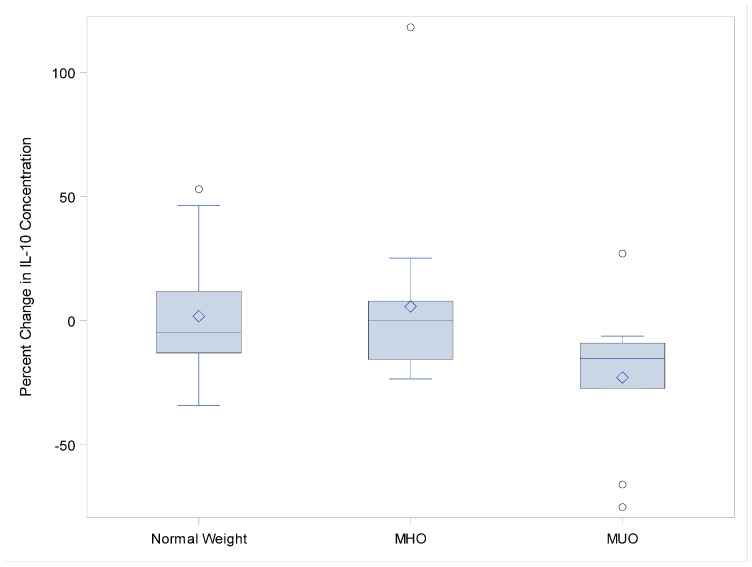
IL-10 percent change in concentration from baseline to 1 month post-influenza vaccination.

**Figure 4:**
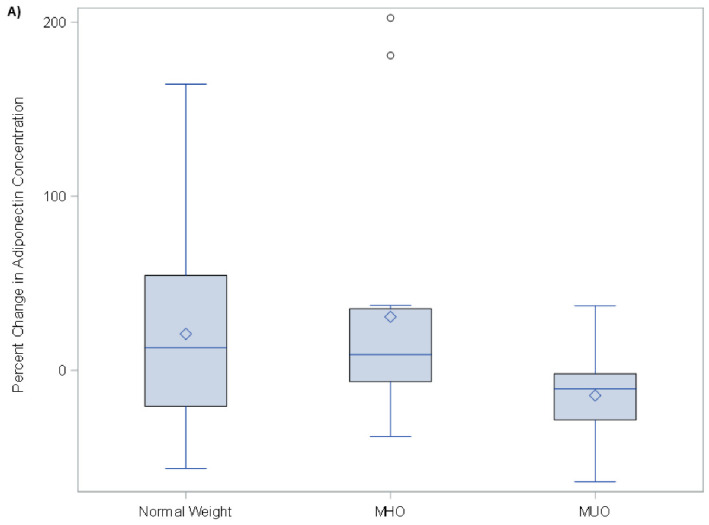

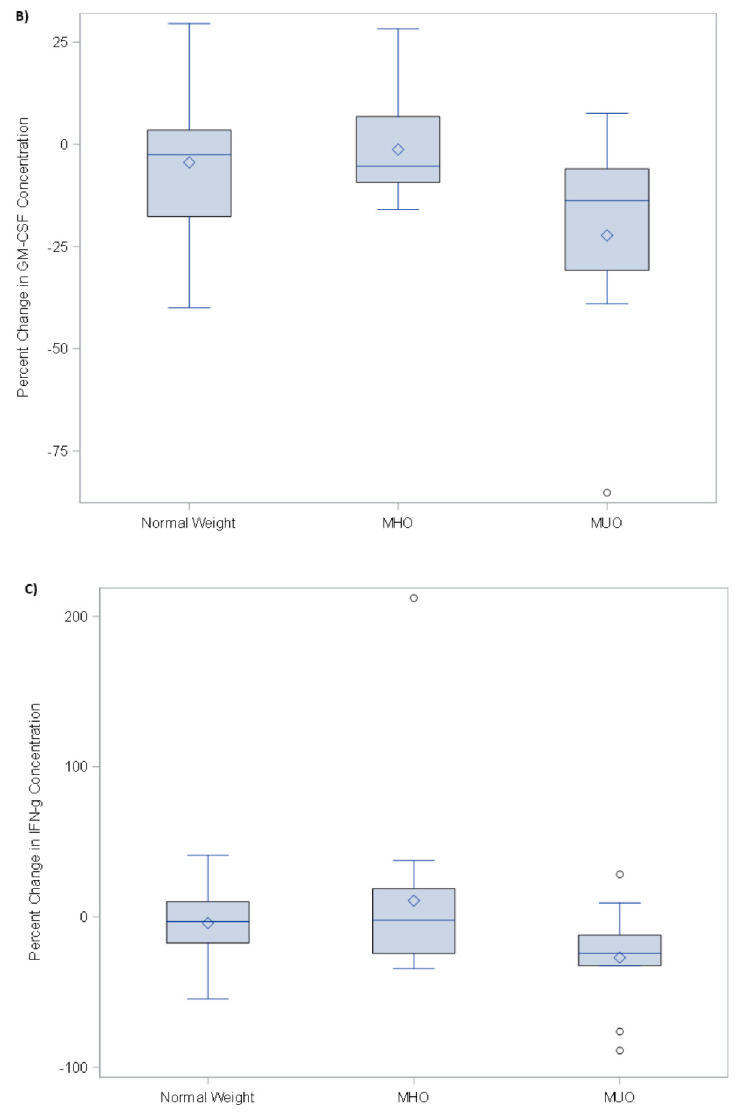
(**A**–**C**) Adiponectin, GM-CSF, and IFN-g percent change from baseline to 1 month post-influenza vaccination.

The vaccination-induced change in the serum concentration of IL-13 was statistically significantly lower (*p* = 0.04) in the MUO group relative to both MHO and normal-weight adolescents (Figure 2). IL-10 approached statistical significance (*p*-value = 0.07) for the same trend (Figure 3), with a decrease in concentration, and there appears to be a trend towards clinical significance for adiponectin, leptin, Granzyme B, GM-CSF, and IFN-g. (Figure 4A–C).

## 4. Discussion

Based on a pilot study of a small sample of patients, humoral immune responses to influenza vaccine in overweight/obese children appear to be similar to responses in normal-weight children; larger sample sizes are required to prove this hypothesis. Among NWC, MHOO and MUOO children, our results did not demonstrate statistically significant differences in baseline and 1-month influenza-specific geometric mean titers, seroprotection, and seroconversion. A larger drop in IL-13 response was noted in MUOO adolescents between baseline and one-month post-influenza vaccine compared to MHOO and NWC, suggesting that there is a yet-unidentified diminished immunologic response in this population.

US data from 2016 indicated that 13.7 million children between the ages of 2 and 19 (18.5% prevalence) years were obese [21]. Studies have suggested that complications or hospitalizations due to the influenza H1N1 pandemic strain were identified in 33% of obese children, which was higher compared to NWC [6]. Health risks reside largely in a subgroup of obese individuals generically referred to as “metabolically unhealthy obese/overweight (MUOO).” The identifying characteristic of these individuals is an altered inflammatory state, and because effective vaccination depends on immune induction, this may potentially influence the immune response. However, data on cytokine responses in overweight and obese children are limited. Eliakim et al. evaluated serum cytokine levels and found that serum IL-6 concentrations in obese children were higher than observed in NWC. Controlling for vaccination status, randomly measured anti-tetanus IgG antibody levels were significantly lower in overweight individuals than in NWC [22].

Obesity is known to impact responses to infection [23]. Several studies have identified obesity as an independent risk factor for hospitalization and mortality in patients with H1N1 influenza virus infection [4,5,24]. Further, there are two studies evaluating trivalent influenza vaccine antibody responses in children with obesity. One found that relative to lean controls, children with obesity had a significantly higher rate of seroconversion 1-month post-vaccination [11]. The second study found no significant relationship between BMI percentile and seroconversion in response to influenza vaccine in children, adolescents, and adults [12]. Serologic responses to influenza vaccination in obese adults are difficult to interpret. A previous evaluation on obesity in adults noted that, 1-month post-vaccination, the increase in the influenza antibody titer response was directly proportional to BMI. By 12 months, however, the pattern was reversed; the drop in antibody titers was greater at higher BMI [11]. The development of more effective vaccination programs for this population requires additional data on influenza vaccine response.

Obesity in children is associated with an increased incidence of risk of high blood pressure and high cholesterol, indicators of cardiovascular disease (CVD) in adult life [25,26,27]. However, not all obese adults have the same risk; a subgroup (10–32%) within this population, the MHOO, do not display metabolic disorders such as insulin resistance, lipid abnormalities, or hypertension, and therefore may appear to have a lower risk of obesity-related complications [23,28,29,30]. We found that IL-13 cytokine responses in MHOO individuals are more akin to a normal-weight host and are not similar to responses in MUOO children, shifting the focus of future studies to non-serologic aspects of adaptive immunity among metabolically unhealthy children [31]. Identifying a decreased IL-13 concentration after influenza vaccination in the MUOO cohort indicates that this Th-2 mediated cytokine, which is implicated in counteracting obesity-associated inflammation in children with asthma, suggests a role in vaccine-mediated activation of antigen-presenting cells [32,33]. With equivocal results for both obesity and metabolic health in terms of humoral immunity, the next steps will be to evaluate influenza-specific T-cell responses or to evaluate other vaccine types, such as mRNA platforms.

This study investigated if obesity in children is associated with more robust markers of inflammation such as vaccine-specific antibody responses such as seroprotection and seroconversion as well as cytokine production, including serum levels of adiponectin and leptin, and whether these markers can be used to differentiate MHOO children more precisely from MUOO children. Identifying additional markers of chronic disease provides a more complete description of the inflammatory profile associated with obesity while determining if these markers can more completely differentiate between MHOO and MUOO individuals and have broad implications beyond the scope of this study.

Limitations to our data include using a narrow definition of metabolic health and a combination of overweight and obese children in one group. More recent data suggests that using HbA1c, CRP, and other potential markers of inflammation in pediatric infections may be indicative of metabolic health [34]. Additionally, we did not further investigate immune or cytokine responses of normal-weight adolescents who may have markers of poor metabolic health. Controlling for previous seasons of influenza vaccination and/or exposure may provide more insight on immune responses in MUOO/MHOO children. Finally, we were limited by sample size due to loss to follow-up/withdrawal and ideally would benefit from the use of a vaccine response that is also implicated in obesity and severe disease, such as SARS-CoV-2.

## 5. Conclusions

Our data provide the impetus to (1) examine alternative approaches to simple vaccination against influenza in overweight and obese children, (2) determine if there are similar responses to vaccination against other micro-organisms, including not just other viruses such as SARS-CoV-2 but also bacteria (for example, pneumococcus) and (3) investigate the specific effects of obesity on both innate and adaptive immune responses. This last would include a search for the genetic and environmental/socioeconomic underpinnings of both obesity and the immune response axis. This investigation opens the question of the importance of the MHOO and MUOO phenotypes as determinants of vaccination responses in children. Our results show that humoral immunity does not drive the MHOO/MUOO dichotomy, providing preliminary data for a host of other investigations where this distinction may be germane. Ultimately, this knowledge may lead to the development of an approach to evaluating the innate arm of immunity and influenza and other vaccine-specific T-cell responses.

## Figures and Tables

**Table 1:** Demographics and metabolic health profiles of patients in cohort with influenza antibody responses and cytokine profiles.

	Antibody Testing (*n* = 43)	Cytokine Testing (*n* = 50)
Age, years, IQR (median)	11.9–15.4 (14.0)	9.6–17.5 (14.0)
Female, *n*, (%)	26 (60.0)	32 (64.0)
Race, *n* (%)		
African American	9 (21.0)	8 (16.3)
Caucasian	10 (23.0)	14 (28.6)
Native American	1 (2.0)	1 (2.0)
Asian	10 (23.0)	10 (20.4)
Other	13 (30.0)	16 (32.7)
Metabolic Health Parameters *n* (%)		
Triglycerides > 110 mg/dL	11 (26.0)	17 (34.0)
HDL < 40 mg/dL	3(7.0)	4 (8.0)
Blood pressure > 90 th percentile	3 (7.0)	3 (6.1)
Glucose > 100 mg/dL	11 (26.0)	13 (25.0)
Waist circumference	13 (30.0)	15 (30.0)
BMI > 85th percentile *n* (%)		23 (46.0)
Metabolically healthy	12 (29.0)	14 (28.0)
Metabolically unhealthy	7 (16.0)	9 (18.0)

**Table 2:** Pre-existing antibody responses before vaccination against influenza A/H1N1 and H3N2 and influenza B/Victoria and Yamagata lineages according to BMI and metabolic status. A total of 58 subjects had serum sampled before vaccination. From these 58 subjects, 35 were classified as BMI normal, 13 subjects as BMI high MHOO (metabolic healthy obese/overweight) and 10 as BMI High MUOO (metabolic unhealthy obese/overweight).

	Influenza A H1N1				Influenza A H3N2				Influenza B Victoria				Influenza B Yamagata			
	GMT_pre_ CI 95%	*p*-Value	Baseline Seroprotection No. (%)	*p*-Value	GMT_pre_ CI 95%	*p*-Value	Baseline Seroprotection No. (%)	*p*-Value	GMT_pre_ CI 95%	*p*-Value	Baseline Seroprotection No. (%)	*p*-Value	GMT_pre_ CI 95%	*p*-Value	Baseline Seroprotection No. (%)	*p*-Value
**BMI Normal** ***n* = 35**	116.5 (73.9–183.9)	0.82	31 (88.6)	0.67	87.9 (55.6-138.9)	0.57	30 (85.7)	0.63	22.8 (15.1–34.4)	0.30	20 (57.1)	0.14	7.4 (5.5–10.1)	0.31	6 (17.1)	0.13
**BMI High MH** ***n*** **= 13**	133.4 (87.3–203.7)		13 (100)		85.1 (36.7–197.8)		11 (84.6)		14.7 (7.4–28.9)		4 (30.8)		6.2 (4.5–8.5)		0 (0)	
**BMI High MU** ***n*** **= 10**	98.5 (58.2–166.5)		9 (90)		139.3 (79.3–244.6)		10 (100)		12.8 (5.2–31.9)		3 (30)		5.0 (5.0–5.0)		0 (0)	

GMTpre: geometric mean titer before vaccination. *p* < 0.05 was considered significantly significant. The GMT and 95% confidence intervals were calculated by taking the exponent (log2) of the mean and of the lower and upper limits of the 95% confidence intervals of the log2-transformed titers. ANOVA test was performed to compare the mean differences of the GMTs within each lineage.

**Table 3:** Antibody responses 25–42 days after vaccination against influenza A/H1N1 and H3N2 and influenza B/Victoria and Yamagata lineages according to BMI and metabolic status. A total of 46 subjects had serum sampled after vaccination. From these 46 subjects, 25 subjects were classified as BMI normal, 12 subjects as BMI high MH (metabolic healthy) and 9 as BMI high MU (metabolic unhealthy).

	Influenza A H1N1				Influenza A H3N2				Influenza B Victoria				Influenza B Yamagata			
	GMT_post_ CI 95%	*p*-Value	Seroprotection No. (%)	*p*-Value	GMT_post_ CI 95%	*p*-Value	Seroprotection No. (%)	*p*-Value	GMT_post_ CI 95%	*p*-Value	Seroprotection No. (%)	*p*-Value	GMT_post_ CI 95%	*p*-Value	Seroprotection No. (%)	*p*-Value
BMI Normal *n* = 25	195.2 (122.9–309.9)	0.81	24 (96)	>0.9	226.8 (177.0–290.6)	0.28	25 (100)	0.20	35.0 (19.5–62.9)	0.89	17 (68)	0.77	10.3 (6.0–17.7)	0.59	6 (24)	0.48
BMI High MH *n* = 12	242.1 (149.4392.4)		12 (100)		131.4 (62.4–276.3)		11 (91.7)		28.3 (12.9–62.0)		7 (58.3)		11.9 (5.1–27.6)		4 (33.3)	
BMI High MU *n* = 9	217.7 (136.1–348.3)		9 (100)		160.0 (48.6–526.6)		8 (88.9)		37.0 (10.2–134.5)		5 (55.6)		17.9 (5.2–61.9)		4 (44.4)	

GMTpost: geometric mean titer after vaccination. *p* < 0.05 was considered significantly significant. The GMT and 95% confidence intervals were calculated by taking the exponent (log2) of the mean and of the lower and upper limits of the 95% confidence intervals of the log2-transformed titers. ANOVA test was performed to compare the mean differences of the GMTs within each lineage.

**Table 4:** Antibody responses 25–42 days after vaccination against influenza A/H1N1cand H3N2 and influenza B/Victoria and Yamagata lineages in the general cohort and according to BMI and metabolic status. A total of 43 patients had complete follow-up and serum samples were collected both before and after vaccination. From these 43 samples, 24 were classified as BMI normal, 12 samples as BMI high MH (metabolic healthy) and 7 samples as BMI high MU (metabolic unhealthy).

	Influenza A H1N1				Influenza A H3N2				Influenza B Victoria				Influenza B Yamagata			
	GMR CI 95%	*p*-Value	Seroconversion No. (%)	*p*-Value	GMR CI 95%	*p*-Value	Seroconversion No. (%)	*p*-Value	GMR CI 95%	*p*-Value	Seroconversion No. (%)	*p*-Value	GMR CI 95%	*p*-Value	Seroconversion No. (%)	*p*-Value
BMI Normal *n* = 24	2.3 (1.5–3.4)	0.80	8 (33.3)	0.74	2.5 (1.5–4.4)	0.51	8 (33.3)	0.73	1.9 (1.3–2.8)	0.62	3 (12.5)	0.27	1.5 (0.8–2.6)	0.31	5 (20.8)	0.47
BMI High MH *n* = 12	2.0 (1.2–3.1)		4 (33.3)		1.7 (0.5-5.5)		3 (25.0)		2.1 (1.0-4.3)		4 (33.3)		1.9 (0.9–4.0)		2 (16.7)	
BMI High MU *n* = 7	1.8 (0.9–3.6)		1 (14.3)		1.3 (0.8–2.2)		1 (14.3)		3.1 (0.6–17.1)		2 (28.6)		3.6 (0.7–17.7)		3 (42.9)	

GMR: geometric mean ratio. *p* ≤ 0.05 was considered significantly significant. The GMR and 95% confidence intervals were calculated by taking the exponent (log2) of the mean and of the lower and upper limits of the 95% confidence intervals of the log2-fold induction transformed titers. ANOVA test was performed to compare the GMR within each lineage. Seroconversion was considered when fold induction of HI values was >4. Seroconversion was analyzed by Chi-square test or Fisher’s exact test, as appropriate, for each lineage.
